# Effects of Leaf Extracts from Genetic Resource of *Capsicum* spp. on Neuroprotection and Anti-Neuroinflammation in HT22 and in BV2 Cells

**DOI:** 10.3390/plants13192820

**Published:** 2024-10-08

**Authors:** Linsha Dong, Bo-Ram Choi, Hyo Bong Jeong, Hwan Lee, Zhiming Liu, Dahye Yoon, Hye Eun Lee, Dong-Sung Lee, Dae Young Lee

**Affiliations:** 1 Research Institute of Pharmaceutical Sciences, College of Pharmacy, Chosun University, 309 Pilmun-daero, Dong-gu, Gwangju 61452, Republic of Korea; donglinsha011@163.com (L.D.); ghksdldi123@hanmail.net (H.L.); lzmqust@126.com (Z.L.); 2 Department of Herbal Crop Research, National Institute of Horticultural and Herbal Science, Rural Development Administration, Eumseong 27709, Republic of Korea; bmcbr@korea.kr (B.-R.C.); dahyeyoon@korea.kr (D.Y.); 3 Department of Horticultural Crop Research, National Institute of Horticultural and Herbal Science, Rural Development Administration, Wanju 55365, Republic of Korea; bong9846@korea.kr (H.B.J.); helee72@korea.kr (H.E.L.); 4 BK21 FOUR KNU Creative BioResearch Group, School of Life Sciences, Kyungpook National University, Daegu 41566, Republic of Korea

**Keywords:** *Capsicum* spp., develop functional varieties, neurodegenerative diseases, HT22, BV2

## Abstract

To develop functional varieties of *Capsicum* spp. leaves, 40 genetic resources were collected and extracted with 30% aqueous-fermented ethanol. We investigated the protective effects of extracts from 40 genetic resources of *Capsicum* spp. on glutamate-induced HT22 and LPS-induced BV2 cells. The results showed that the five extracts exhibited cell-protective activities. We also investigated the anti-inflammatory effects of these five extracts on LPS-induced BV2 cell neuroinflammation and found that 23OM18 exhibited superior anti-inflammatory effects. We further investigated the protective activity and anti-inflammatory mechanisms of 23OM18 in these two cell models. In addition, the profiles of 16 metabolites were compared between the representative accessions and among the five genetic resources using ultra-high-performance liquid chromatography quadrupole time-of-flight mass spectrometry (UHPLC-QTOF-MS). The results showed that 23OM18 protected HT22 cells by inhibiting reactive oxygen species generation and regulating the MAPK-JNK signaling pathway, thereby reducing LPS-induced BV2 cell neuroinflammation by regulating the NF-κB and MAPK signaling pathways. Based on these results, 23OM18 has the potential to be developed as a functional food for the treatment of neurodegenerative diseases.

## 1. Introduction

Neurodegenerative diseases (NDs) are gradually becoming a major burden on human society, affecting millions of people worldwide. The resulting incidence and mortality rates are major healthcare issues. NDs are a diverse category of complicated illnesses characterized by neuronal loss and the gradual degeneration of several parts of the nervous system. Alzheimer’s disease, Parkinson’s disease, Huntington’s disease, and amyotrophic lateral sclerosis are the main types of NDs [1,2]. NDs have complicated and varied pathophysiologies that often include oxidative stress, mitochondrial dysfunction, impaired metal homeostasis, and neuro-inflammation. The molecular mechanism of neurodegeneration remains unknown and presents a significant challenge in the development of effective therapies [3]. Therefore, our understanding of the molecular underpinnings of the neurodegenerative processes must be improved to help discover therapeutic targets for therapy and prevention.

The identification of innate immune function-related AD risk genes and higher levels of inflammatory markers in patients with AD suggest that neuro-inflammation is a major factor in the pathophysiology of AD [4]. Throughout the central nervous system, microglia, which are resident macrophages of the central nervous system, constitute a vast network that helps maintain homeostasis, monitor pathogens and cell debris, and participate in the response and repair processes. Microglia in NDs help with debris clearing but can also contribute to pathogenesis. Microglial damage may be the basis of NDs [5]. Early inflammation following CNS injury is advantageous for eliminating pathogens and delaying the onset of damage, both of which promote tissue healing and regeneration. However, chronic neuro-inflammation associated with neurological disorders may affect the nervous system. Activated microglia boost their phagocytic activity and release pro-inflammatory mediators, such as nitrogen oxide (NO), reactive oxygen species (ROS), cytokines, and chemokines, in response to pathogenic infections [6]. Activated microglia not only play a role in the defense and removal of pathogens but also lead to harm to the brain tissue and cells. They serve as primary initiators of the inflammatory cascade through an increase in chemokine production and responsiveness.

An imbalance in oxidative stress is the primary cause of neurodegenerative illnesses. Neuronal cells are highly vulnerable to oxidative stress and are likely to produce significant quantities of ROS because of their high oxygen consumption [7,8]. Glutamate is the primary mediator of the excessive excitatory neurotransmission induced by excitatory toxicity in neurons. In the hippocampal region, elevated levels of extracellular glutamate stimulate N-methyl-d-aspartate receptors, facilitating the uptake of Ca^2+^ into neuronal cells. Increased intracellular Ca^2+^ levels cause ROS generation, and when these ROS accumulate, they can oxidize proteins, lipids, and DNA, causing damage to cells [9].

*Capsicum* spp., commonly known as chili peppers, belong to the family Solanaceae. Dried chili pepper has been used in the human diet as an important spice and source of nutrients. *Capsicum* spp. are also used as raw materials in several traditional drug therapies. The fruits of *Capsicum* spp. have been used to cure dyspepsia, appetite, and flatulence, as well as to enhance digestion and circulation. They have also been used as tonics and antibacterial and stimulatory agents [10]. Chili peppers are nutritious, functional foods that contain numerous healthy chemicals, including carotenoids, anthocyanins, flavonoids, phenolic compounds, capsaicin, and various vitamins and minerals [11]. However, the health benefits of chili peppers on degenerative diseases have not yet been studied. In this study, 40 genetic resources were collected, including those with high alpha-glucosidase inhibitory (AGI) activity and high content of major flavonoid components. We investigated the protective effects of extracts from 40 genetic resources of *Capsicum* spp. on glutamate-induced HT22 and LPS-induced BV2 cells. The results have potential applications in various industries, including food, nutraceuticals, and pharmaceuticals.

## 2. Results

### 2.1. Passport Data of 40 Generic Resources from Capsicum spp.

Information regarding the source of introduction and the species name of each genetic resource is summarized in Table 1. Most accessions belonged to the cultivated species *Capsicum* annuum; however, some resources belonged to other species with different morphological and physiological characteristics. Table 2 shows the key morphological characteristics of the genetic resources, including the plant height and leaf length. The average plant height of the genetic resources was 73.0 cm, and the average leaf length was 11.9 cm. 23OM18 was taller than average, but the length of the leaves was similar.

### 2.2. Effects of Leaf Samples of the 40 Genetic Resources from Capsicum spp. Extracts on Glutamate-Induced Oxidative Stress in HT22 Cells

**Figure 1:**
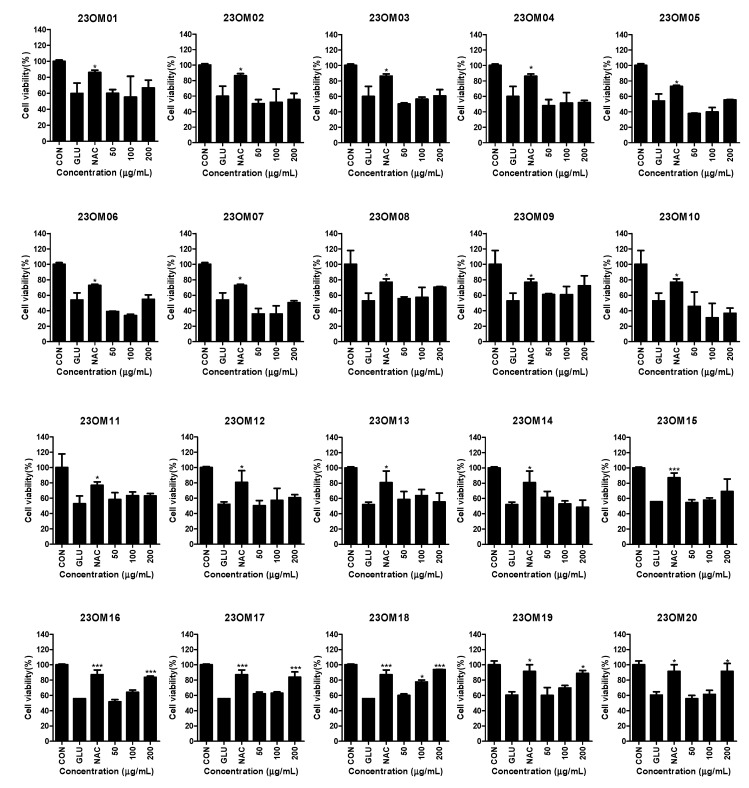

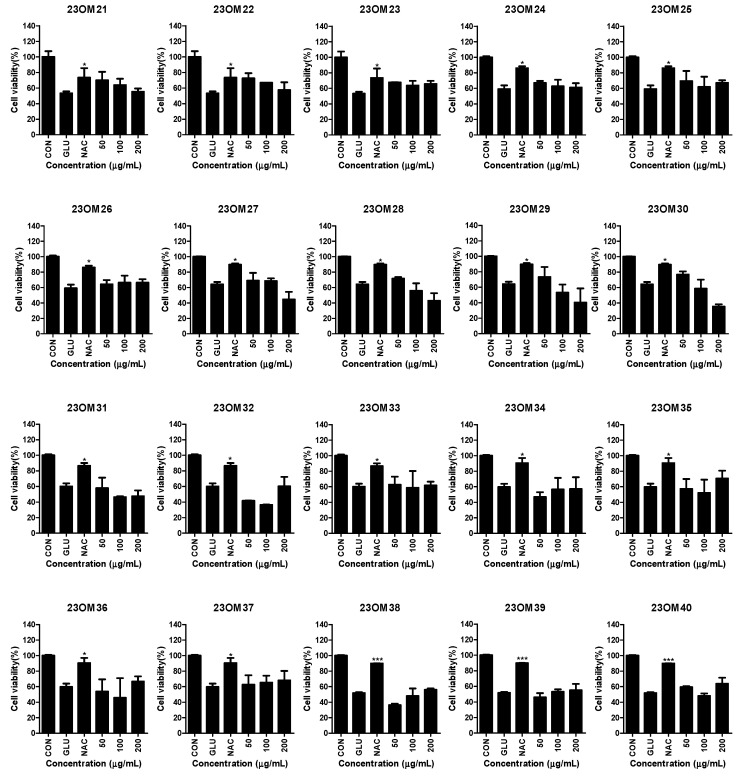
Protective effects of the 40 genetic resources from *Capsicum* annuum extracts on glutamate-induced HT22 cells. HT22 cells were pre-treated with 23OM01–40 (50–200 μg/mL) for 3 h and then stimulated with glutamate (5 mM) for 24 h. Cell viability was then detected. Data are presented as the mean ± standard deviation (*n* = 3). * *p* < 0.05; *** *p* < 0.001 compared with the glutamate group. N-acetyl-cysteine (NAC, 1 mM) was used as a positive control.

To explore whether these 40 extracts have neuroprotective effects, we used a glutamate-induced HT22 cell model to screen all the extracts. Among these 40 extracts, 5 extracts (23OM16, 23OM17, 23OM18, 23OM19, and 23OM20) showed significant protective effects against glutamate-induced cell death (Figure 1). The cell protection values (EC_50_) are shown in Table 3. Of these, 23OM18 exhibited the highest protective activity with an EC_50_ value of 102.6 ± 0.2934 μg/mL.

### 2.3. Effects of 23OM18 on ROS Production in HT22 Cells

**Figure 2:**
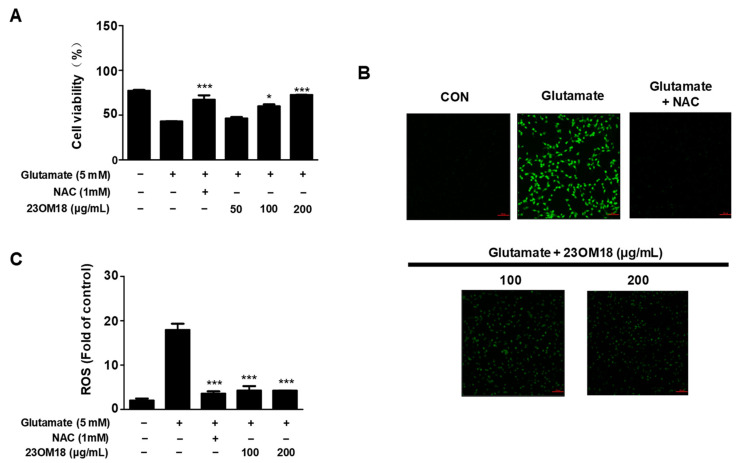
Protective effect of 23OM18 on glutamate-induced HT22 cells (**A**), and ROS production (**B**,**C**). Cells were pre-treated with 23OM18 at concentrations of 50–200 μg/mL for 3 h, followed by stimulation with glutamate (5 mM) for 24 h. Data are presented as the mean ± standard deviation (*n* = 3). * *p* < 0.05; *** *p* < 0.001 compared with the glutamate-treated group.

After stimulation with glutamate for 8 h, we examined ROS production in HT22 cells using DCFH-DA and photographed them under a fluorescence microscope. Due to the lack of protective effect at 50 μg/mL, we performed the ROS experiment only at 100 and 200 μg/mL concentrations of 23OM18. As shown in Figure 2B,C, 23OM18 at concentrations of 100 and 200 μg/mL significantly reduced ROS generation.

### 2.4. Effects of 23OM18 on the MAPK Signaling Pathway in HT22 Cells

**Figure 3:**
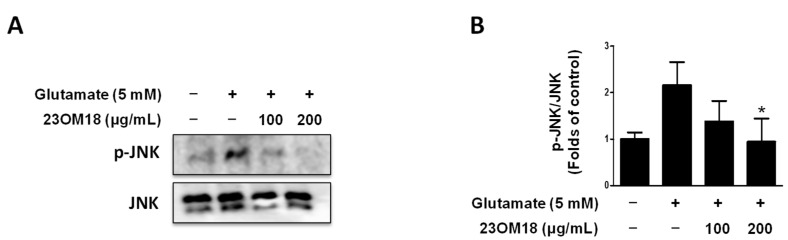
Effects of 23OM18 on the MAPK-JNK signaling pathway in glutamate-induced HT22 cells (**A**,**B**). Cells were pre-treated with 23OM18 at concentrations of 100–200 μg/mL for 3 h, followed by stimulation with glutamate (5 mM) for 8 h. Data are presented as the mean ± standard deviation (*n* = 3). * *p* < 0.05 compared with the glutamate-treated group.

To explore the mechanism by which 23OM18 exerts its protective effect, we examined the effect of 23OM18 on the MAPK signaling pathway. We found that 23OM18 inhibited JNK phosphorylation. The results are shown in Figure 3 A,B. In the MAPK pathway, p38 and ERK levels were not affected by 23OM18.

### 2.5. Effects of 23OM18 on LPS-Induced Cytokine Secretion in BV2 Cells

**Figure 4:**
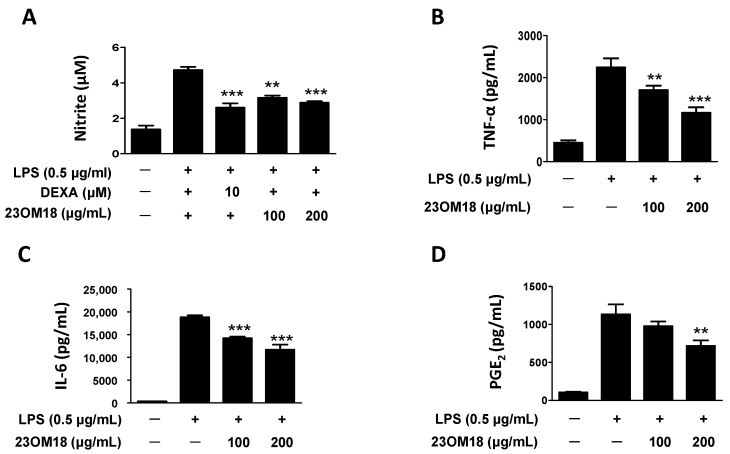
Inhibitory effects of 23OM18 on LPS-induced cytokine secretion in BV2 cells: nitrite (**A**), TNF-α (**B**), IL-6 (**C**), and PGE2 (**D**). Cells were pre-treated with 23OM18 at concentrations of 100–200 μg/mL for 3 h, followed by stimulation with LPS (0.5 μg/mL) for 24 h. Data are presented as the mean ± standard deviation (*n* = 3). ** *p* < 0.01 *** *p* < 0.001 compared with the LPS-treated group. Dexamethasone (DEXA) was used as a positive control.

In previous experiments, we found that 23OM18 has a neuroprotective effect on hippocampal neuronal cells. We explored the effects of 23OM18 on LPS-induced BV2 cells. BV2 cells were pre-treated with 23OM18 for 3 h and then stimulated with LPS for 24 h. The levels of pro-inflammatory factors in the cell supernatants were then determined. The results are shown in Figure 4. The levels of nitrite, TNF-α, IL-6, and PGE_2_ were significantly increased by LPS. The 23OM18-treated group exhibited inhibitory effects on these pro-inflammatory mediators.

### 2.6. Effects of 23OM18 on iNOS and COX-2 Expression in LPS-Induced BV2 Cells

**Figure 5:**
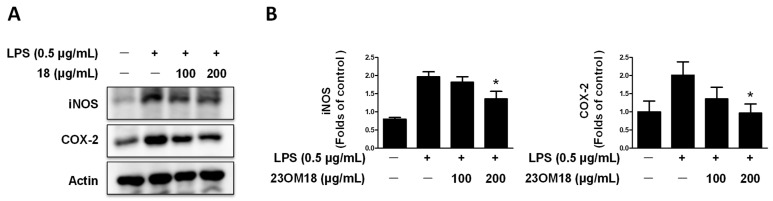
Effects of 23OM18 on LPS-induced iNOS and COX-2 expression on BV2 cells (**A**,**B**). Cells were pre-treated with 23OM18 at concentrations of 100–200 μg/mL for 3 h, followed by stimulation with LPS (0.5 μg/mL) for 24 h. Data are presented as the mean ± standard deviation (*n* = 3). * *p* < 0.05 compared with the LPS-treated group.

The expression of inducible nitric oxide synthase (iNOS), the enzyme that catalyzes the production of NO from L-arginine, is frequently associated with inflammatory and malignant diseases. The expression of iNOS was significantly increased by LPS. As shown in Figure 5A,B, pre-treatment with 23OM18 inhibited iNOS expression. Another enzyme that plays an important role in inflammation is COX-2. COX-2 catalyzes the rate-limiting step in the conversion of arachidonic acid into prostaglandins. The initial inflammatory response to injury or infection is strongly induced by COX-2, which is seldom detectable under normal physiological conditions. In our results, 23OM18 also decreased the expression of COX-2. The results are shown in Figure 5A,B. 23OM18 has a significant inhibitory effect on the expression of iNOS and COX-2. It also exerts anti-inflammatory effects.

### 2.7. Effects of 23OM18 on the NF-κB Signaling Pathway in LPS-Induced BV2 Cells

**Figure 6:**
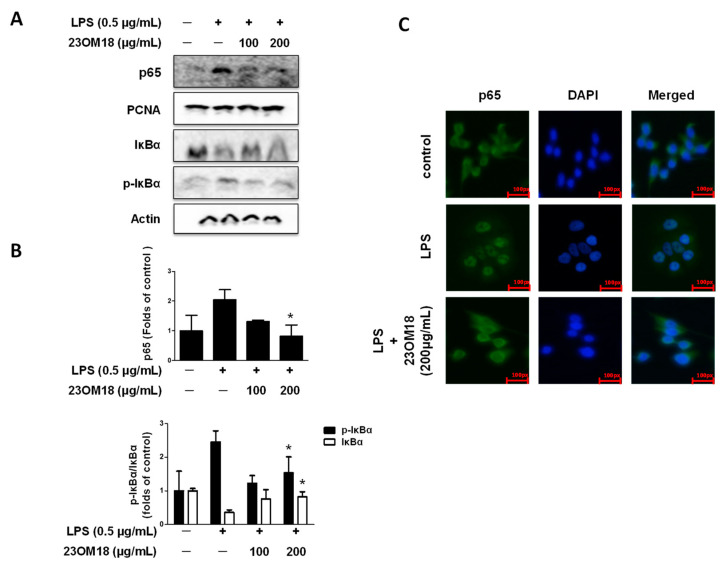
Effects of 23OM18 on the NF-κB signaling pathway on LPS-induced BV2 cells (**A**–**C**). Cells were pre-treated with 23OM18 at concentrations of 100–200 μg/mL for 3 h, followed by stimulation with LPS (0.5 μg/mL) for 15 min. Data are presented as the mean ± standard deviation (*n* = 3). * *p* < 0.05 compared with the LPS-treated group.

NF-κB signaling is critical for immune responses. Normally, NF-κB forms a complex with inhibitory IκB proteins in the cytoplasm, where it is sequestered in an inactive state. These signals, which are triggered by stimuli, target NF-κB complexes and translocate them to the nucleus, where they control gene transcription. We examined the regulatory effects of 23OM18 on the NF-κB signaling pathway. As shown in Figure 6A,B, the transfer of NF-κB (p65) into the nucleus caused by LPS stimulation was significantly inhibited by 23OM18, which also showed an inhibitory effect on the phosphorylation of IκBα and inhibited the decrease in IκBα expression.

Immunofluorescence for p65 is shown in Figure 6C. The translocation of p65 into the nucleus, induced by LPS, was significantly inhibited by 23OM18. Thus, 23OM18 has a significant regulatory effect on the LPS-induced activation of the NF-κB signaling pathway.

### 2.8. Effects of 23OM18 on the MAPK Signaling Pathway in LPS-Induced BV2 Cells

**Figure 7:**
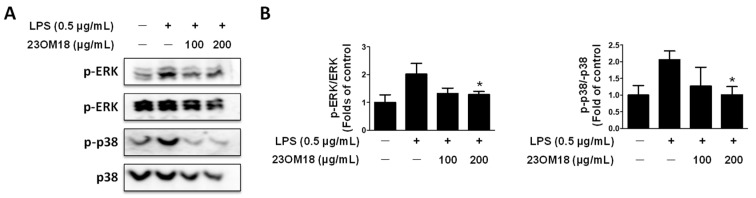
Effects of 23OM18 on the MAPK signaling pathway in LPS-induced BV2 cells (**A**,**B**). Cells were pre-treated with 23OM18 at concentrations of 100–200 μg/mL for 3 h, followed by stimulation with LPS for 30 min. Data are presented as the mean ± standard deviation (*n* = 3). * *p* < 0.05 compared with the glutamate-treated group.

The process of inflammatory activation usually involves more than one signaling pathway. After being phosphorylated, MAPKs proceed to phosphorylate and activate transcription factors located in the nucleus or cytoplasm, which results in the activation of target genes and thus initiates a range of biological reactions. Western blot results are shown in Figure 7A,B. 23OM18 had a significant inhibitory effect on the expression of phosphorylated ERK and p38 but had no effect on phosphorylated JNK. These results indicate that 23OM18 has a regulatory effect on the MAPK signaling pathway.

### 2.9. Effects of 23OM18 on LPS-Induced BV2 Cells Co-Cultured with HT22 Cells

**Figure 8:**
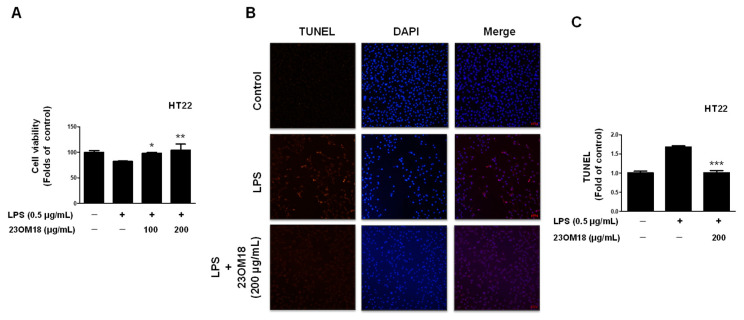
Protective effects of 23OM18 on LPS-induced BV2 cells co-cultured with HT22 cells (**A**–**C**). In the BV2-HT22 co-culture system, BV2 cells were pre-treated with 23OM18 at concentrations of 100–200 μg/mL for 3 h, followed by stimulation with LPS for 24 h. The viability of HT22 cells was assessed. Data are presented as the mean ± standard deviation (*n* = 3). * *p* < 0.05; ** *p* < 0.01; *** *p* < 0.001, compared with the LPS-treated group.

To develop an in vitro neuro-inflammation model by establishing a microglia–neuron co-culture system, BV2 microglia were seeded into the upper chamber of the trans-well plate, and HT22 cells were seeded into the lower chamber to establish a BV2-HT22 cell co-culture system. BV2 cells in the upper chamber were stimulated with LPS, and their effect on HT22 neurons in the lower chamber was observed. As shown in Figure 8A, LPS-induced BV2 cells caused the partial death of HT22 cells in the lower chamber, while 23OM18 exerted a protective effect on the HT22 cells. TUNEL staining was performed on HT22 cells, and red-positive cells were observed (Figure 8B,C). This indicated that excessive neuro-inflammation can cause neuronal apoptosis in the BV2-HT22 co-culture system. 23OM18 also plays a role in protecting neurons in co-culture systems.

### 2.10. Secondary Metabolite Profiling of 23OM16–23OM20

To compare the five extracts (23OM16–23OM20) that showed significant protective effects against glutamate-induced cell death, secondary metabolite profiling was performed using UPLC-QTOF/MS. Table 4 presents the results of the study. Flavonoids such as apigenin and luteolin, organic acids such as citric acid and quinic acid, and capsianoside were detected. Interestingly, capsianosides 1 and 2 were detected at higher levels in 23OM18, which showed the best protective activity compared to other extracts. These results suggested that capsianoside and its derivatives may be involved in the protective activity of 23OM18.

## 3. Discussion

Oxidative stress (OS) is a critical factor in the development of various pathophysiological conditions. ROS and reactive nitrogen species (RNS) are associated with oxidative stress. They are involved in signaling and preserving physiological homeostasis at appropriate levels [12,13]. These substances are highly destructive to biological molecules, damage DNA, proteins, and lipids, and are often associated with various diseases, including cancer, inflammation, and NDs [14]. Glutamate is the primary mediator of excitatory neurotransmission in the brain [15]. In the nervous system, glutamate is associated with key aspects of normal brain function, including synaptogenesis, learning, cognition, and memory. High glutamate concentrations can induce excessive neuronal depolarization and calcium influx in some illnesses, which can result in oxidative stress, mitochondrial instability, and activation of pathways that ultimately lead to cell death [16]. High amounts of extracellular glutamate deplete intracellular cystine and glutathione, which inhibits cystine absorption into cells via the cystine/glutamate antiporter system and is the mechanism underlying oxidative glutamate toxicity. Oxidative stress is caused by an excessive build-up of reactive oxygen species (ROS) as a result of glutathione depletion [17]. HT22 cells are well-known mouse-derived hippocampal cells. BV2 cells, as microglia, are recognized for their role in regulating neuro-inflammation. Therefore, we aimed to investigate the protective effects in an oxidative damage model induced by glutamate in HT22 hippocampal cells and the modulatory effects on neurodegenerative diseases in BV2 microglia using a neuro-inflammation model induced by LPS in this study. Initially, we evaluated the cytotoxicity of all 40 extracts by treating HT22 cells with concentrations up to 200 µg/mL. As a result, 23OM10 and 23OM30 exhibited cytotoxicity at the highest concentration of 200 µg/mL, whereas all other extracts showed no cytotoxicity at concentrations up to 200 µg/mL. In addition, 23OM18 showed the highest significantly protective effects against glutamate-treated HT22 cells. ROS fluorescence results indicated that the green fluorescence of ROS in HT22 cells pre-treated with 23OM18 was significantly weakened.

MAPK is a kinase cascade that plays an important role in the cellular responses to oxidative stress and inflammation. They can activate MAPK through multiple mechanisms: ROS are directly activated, calcium ions flow into the cells, and free radicals directly oxidize the enzymes in the pathway. ERK is an extracellular kinase that plays a role in cell proliferation, differentiation, and survival. JNK is a c-Jun N-terminal kinase that plays an important role in apoptosis and inflammation. p38 kinase plays a role in cell survival, inflammation, and oxidative stress responses [18,19]. In LPS-induced BV2 cells and glutamate-induced HT22 cells, we detected the regulatory effects of 23OM18 on the MAPK signaling pathway. In HT22 neurons, 23OM18 inhibited glutamate-induced phosphorylation of JNK. Simultaneously, in BV2 microglia, 23OM18 inhibited the phosphorylation of ERK and p38. This evidence indicates that 23OM18 can regulate the MAPK signaling pathway, which may be the basis for its anti-inflammatory and neuroprotective effects.

Increasing evidence supports the fundamental role of neuro-inflammation in the development of NDs. Pro-inflammatory cytokines play a major role in triggering inflammatory responses in the brain [20]. Overactivated microglia will undergo significant changes in phenotype and morphology, accompanied by increased expression of the pro-inflammatory cytokines TNFα, IL-6, IL-1β, and NO. This aggressive state of microglia leads to the formation of a chronic neuro-inflammatory environment and exacerbates neuronal and synaptic loss [21]. In the present study, we detected representative pro-inflammatory cytokines in the activated microglia, such as nitrite, TNF-α, IL-6, and PGE2. These factors play distinct roles in inflammation development. Excessive production of TNF-α can induce inflammatory genes, cell death, endothelial upregulation, recruitment, and the activation of immune cells [22]. As a soluble mediator, IL-6 exerts pleiotropic effects on immunological responses, hematopoiesis, and inflammation. Continued dysregulation of IL-6 production leads to the development of various diseases [23]. When pro-inflammatory cytokines or bacterial products induce immune or epithelial cells, induced NOS (iNOS) is produced. After expression, iNOS continues to generate a large amount of NO for a long time. Higher quantities of NO and its by-products can be harmful to organisms in a number of ways, including blocking important enzymes required for respiration and reproduction [24]. Prostaglandin E2, one of the most prevalent prostaglandins in the body, is connected to a number of inflammatory and immunological processes. It increases vascular permeability and enhances edema and leukocyte infiltration. 23OM18 inhibits the release of these pro-inflammatory factors in LPS-induced microglia, which may be the basis for 23OM18’s regulatory effect on neuro-inflammation.

A bidirectional conversation exists between neurons and microglia in the internal environment of the brain, which is important for steady-state function and is linked to NDs that impair cognitive function [25]. Sustained microglial activation triggers a chronic neuro-inflammatory response that disrupts neuronal health and communication between neurons and microglia [26]. In this study, we established an in vitro co-culture model of hippocampal neurons (HT22) and microglia (BV2) to evaluate the effects of 23OM18 on LPS-induced signaling pathways in microglia and cell viability of hippocampal neurons. Partial apoptosis of HT22 neuronal cells was observed when they were co-cultured with LPS-induced BV2 microglia. 23OM18 exerted a protective effect against HT22 cells. These results indicate that *C. annuum* can exert neuroprotective effects by regulating neuro-inflammation in microglia and reducing neuro-inflammatory damage to neurons.

Secondary metabolite analysis of *C. annuum* leaves showed high protective activity, and various compounds such as flavonoids, organic acids, and capsianoside were detected. Among them, higher contents of capsicnoside II and the new capsianoside III were detected in 23OM18, which exhibited the best protective activity. The capsianoside group is an acyclic diterpene glycoside specific to the genus *Capsicum*. It is classified among monomeric diterpene glycosides, including capsianosides I–XVII, or dimeric esters, including capsianosides A–L [27]. Most of these are found in fruits but have also been reported to be detected or derived from the leaves of the *Capsicum* genus [28,29].

Although not identified in the secondary metabolite analysis of our study, capsaicin, known as the main component in peppers, is called a capsaicinoid along with dihydro-capsaicin, nordihydrocapsaicin, homodihydrocapsaicin, and homocapsaicin [30]. A study has reported that capsaicin attenuates ROS production in BV2 cells treated with apolipoprotein E, which is known as a genetic risk factor for neurodegenerative diseases [31]. In addition, in a previous study using pepper seed extract, intracellular ROS production in HT22 cells was markedly attenuated with pre-treatment of pepper extract. Luteolin is the bioactive compound in *C. annuum* corresponding to its anti-neuroexcitotoxic effects, and its potential mechanism may involve improving ROS-mediated cell death. However, capsaicin did not show a significant pharmacological effect against neuroexcitotoxicity [32]. In addition, while the majority of previous studies suggest that *C. annuum* and its bioactive compounds are generally safe, prolonged or high-dose exposure has been shown to cause significant mucosal irritation. The primary active compound in *C. annuum*, capsaicin, has reported oral LD50 values of 161.2 mg/kg in rats and 118.8 mg/kg in mice in in vivo models [33].

Excessive ROS cause neuro-oxidative stress, so excessive ROS are associated with a decline in cognitive function [34]. Excessive accumulation of ROS leads to cell death, and the cytoprotective effect in the hippocampal cell line HT-22 is also mainly due to its protective effects against ROS [35]. Additionally, ROS increase the expression of pro-inflammatory genes and promotes the formation and induction of neurotoxicity through lipid peroxidation [36]. In a previous study on capsianosides, compounds isolated from red pepper paste were found to inhibit the formation of CE-OOH, a cholesteryl ester hydroperoxide produced by copper ion-induced oxidation. These compounds were identified as capsianosides F and XVIII, and both showed antioxidant activity by inhibiting CE-OOH formation [37]. In another study, capsianoside V showed the highest DPPH radical activity among the compounds isolated from *Murraya koenigii* [38]. In a previous study using hot peppers, six compounds were isolated, two of which were confirmed to be members of the capsianoside family. These compounds exhibit inhibitory activity against lipid peroxidation [39]. Additionally, these compounds inhibited COX-1 expression by more than 70%. COX-1 causes cognitive deficits and is associated with neuro-inflammation and neuronal death [40]. Based on the results of previous studies, the superior protective activity of 23OM-18 was assumed to be due to its antioxidant activity against ROS.

Nonetheless, we acknowledge that this study has several limitations. First, additional efficacy validation and mechanistic studies are necessary using in vivo models. Furthermore, it is crucial to elucidate the factors contributing to the differences in efficacy among species, as well as to conduct an analysis of the major components present in the most effective extract, 23OM18. We plan to investigate these aspects in future research.

## 4. Materials and Methods

### 4.1. Chemicals and Reagents

Cell culture reagents were purchased from Gibco (Grand Island, NY, USA), and ELISA kits were purchased from BioLegend (San Diego, CA, USA). Primary and secondary antibodies were purchased from Cell Signaling Technology (Danvers, MA, USA). The TUNEL Red Staining Kit was purchased from ROCHE (Basel, Switzerland). All other reagents were purchased from Sigma-Aldrich (St. Louis, MO, USA).

### 4.2. Plant Materials and Extractions

Forty pepper species, including *Capsicum annuum*, *C. chinense*, and *C. frutescens*, were used in this study (Table 1). Most accessions were *C. annuum*, comprising 35 accessions. Their taxonomical information was referenced in the database provided by the National Institute of Agricultural Sciences (NIAS, Jeonju, Republic of Korea) Genebank, the national genetic resources control tower. Along with species information, several key horticultural characteristics corresponding to the voucher can be obtained based on the introduction (IT) number in the Genebank. Species name of accessions without IT numbers were identified based on morphological and genetic characteristics according to previous studies [41]. Seeds of 33 accessions were obtained from the National Institute of Horticultural and Herbal Science (NIHHS, Wanju, Republic of Korea), 11 of which were first introduced to the Genebank. Seeds of the remaining seven cultivars were obtained from the respective seed companies. Accessions up to number 20 were selected based on a previous study on flavonoid metabolite profiling [42]. Seeds were sown on 22 February 2023, in plastic trays (Heungnong, Seoul, Republic of Korea) filled with coco peat-containing bed soil. On 25 April 2023, fully grown 60-day-old seedlings were transferred to a greenhouse using a randomized complete block design. The soil pH and EC were 7.0 and 2.5 dS/m, respectively. The plants were watered regularly using a drip irrigation system and fertilized weekly with a nutrient solution (Daeyu, Seoul, Republic of Korea). At 45 days after transplantation, when the plants were 70 cm tall on average, the fully developed leaves around the buds were harvested for further analysis (Table 2).

The harvested leaves were lyophilized to eliminate residual water and then homogenized. The powdered leaves were extracted with 30% aqueous fermented ethanol under reflux conditions at 70 °C for 4 h, and the residue was subsequently re-extracted under the same conditions. The combined filtrates from the primary and secondary extractions were completely desiccated using a rotary evaporator and used for subsequent experiments.

### 4.3. Cell Culture

HT22 cells were cultured in Dulbecco’s modified Eagle medium (DMEM) (10% FBS), and BV2 cells were cultured in RPMI-1640 medium (10% FBS) under 5% CO_2_ and 37 °C.

### 4.4. ROS Staining

HT22 cells were seeded at a density of 1 × 10^5^ cells/mL in 6-well plates. The cells were pre-treated with 23OM18 at a concentration of 100–200 μg/mL for 3 h. The cells were then stimulated with glutamate (5 μM) for 8 h, the culture medium was discarded, and the cells were washed twice with PBS. The cells were then co-incubated with 10 μM DCFH-DA (FBS-free medium) for 30 min. The cells were washed twice with PBS and photographed under a fluorescence microscope at excitation and emission wavelengths of 488 and 525 nm, respectively.

### 4.5. Western Blot

The harvested cells were lysed with RIPA buffer (St. Louis, MO, USA). To detect the protein concentration, the cells were mixed with the sample buffer for denaturation, followed by SDS-PAGE gel electrophoresis, and then transferred to a nitrocellulose membrane. The membrane was blocked with 5% skim milk (in TBST) for 1 h in room temperature, and incubated with specific primary (1:1000 in 3% skim milk in TBST) overnight at 4 °C, then washed with TBST for 3 times in 30 min, incubated with secondary antibodies (1:5000 in 1% skim milk in TBST) for 1 h in room temperature, washed with TBST for 3 times in 30 min, developed with ECL solution, and photographed.

### 4.6. Nitrite Detection and ELISA

The BV2 cells were seeded at a density of 5 × 10^4^ cells/mL in a 24-well plate. The cells were pre-treated with 100–200 μg/mL 23OM18 for 3 h and then stimulated with LPS (0.5 μg/mL) for 24 h. The cell culture supernatant was preserved. Nitrite detection was performed using the Griess reagent (St. Louis, MO, USA). Griess mixture was mixed with the same volume of the cell supernatant, and absorbance was measured at 570 nM. IL-6, TNF-α, and PGE2 were detected using the ELISA kit with the cell supernatant, following the manufacturer’s instructions.

### 4.7. Protein Extraction

For NF-κB, nuclear and cytoplasmic protein extraction was performed according to the manufacturer’s instructions.

### 4.8. Immunofluorescence

To assess NF-κB (p65) translocation to the nucleus, the BV2 cells were seeded on a round glass slide. The cells were pre-treated with 23OM18 (200 μg/mL) for 3 h and then stimulated with LPS for 15 min. The cells were fixed, solubilized, blocked, incubated with primary antibodies, and then incubated with FITC-linked secondary antibodies. The slides were mounted using an anti-fade mounting medium and photographed under a fluorescence microscope.

### 4.9. BV2-HT22 Co-Culture System

BV2 cells were seeded at a density of 5 × 10^4^ cells/mL in a 24-well plate with a trans-well insert (0.4 μm) in each well above the HT22 cells. The cells were co-cultured for 24 h, pre-treated with 23OM18 (100–200 μg/mL) for 3 h, and then stimulated LPS (0.5 μg/mL) for 24 h. MTT (5 mg/mL) was added to the HT22 culture medium for 1 h, and DMSO was used to dissolve the formazan crystals. The absorbance was read at 540 nm.

### 4.10. TUNEL Red Staining

After stimulation with LPS for 24 h in the BV2-HT22 co-culture system, HT22 cells were used to check for in situ cell death (TUNEL kit), following the manufacturer’s instructions.

### 4.11. Statistical Analysis

Data are presented as the mean ± standard deviation (SD). One-way analysis of variance (ANOVA) followed by Tukey’s post hoc test was performed using GraphPad Prism software (version 8.0). Statistical significance was set at * *p* < 0.05, ** *p* < 0.01, and *** *p* < 0.001. Each experiment was repeated thrice.

### 4.12. Metabolomic Analysis of Plant Extracts Using UPLC-QTOF/MS

For the ultra-high-performance liquid chromatography quadrupole time-of-flight mass spectrometry (UPLC-QTOF/MS) analysis, 100 mg of fine powder was weighed, suspended in 1 mL of solvent (70% MeOH), and ultrasonically extracted for 30 min at room temperature. The supernatant was obtained from samples by centrifugation (13,500× *g*, 5 min at 4 °C). All samples were diluted 20 times and filtered through a syringe filter (PTFE, 0.2 µm). UPLC-QTOF/MS analysis was performed using an ACQUITY-H-Class UPLC system (Waters Corp., Milford, MA, USA). Separation was performed using an Acquity BEH C18 column (2.1 mm × 100 mm; 1.7 um). The column was maintained at 25 °C, whereas the auto sampler was set at 10 °C. The mobile phase consisted of a combination of solvent A (water containing 0.1% formic acid (*v*/*v*)) and solvent B (acetonitrile containing 0.1% formic acid (*v*/*v*)) at a flow rate of 0.4 mL/min. The elution conditions were as follows: 0–4 min, B 10–30%; 4–7 min, B 30–35%; 7–9 min, B maintains 35%; 9–12 min, B 35–40%; 12–15 min, B 40–85%; 15–18 min, B 85–10%. MS analysis was conducted using a Waters Xevo G2-S QTOF MS (Waters Corp.) in the negative ion mode. The MS data were acquired in the MSE acquisition mode, which performs alternate high- and low-energy scans. The operating MS parameters were set as follows: source temperature: 120 °C, desolvation temperature: 550 °C, cone gas flow: 30 L/h, gas flow: 800 L/h, capillary voltage: 2.2 kV, cone voltage: 40 V. Leucine encephalin (*m*/*z* 554.262) was infused during data acquisition as an internal reference to obtain accurate mass measurements.

## 5. Conclusions

NDs are major health issues in the elderly population. We investigated the effects of *Capsicum* spp. leaf samples, obtained from 40 genetic resources from *Capsicum* spp., on NDs. The experimental results indicate that the leaves of the pepper variety 23OM18 (Uiryeonggabeul) exhibit inhibitory effects on neuro-inflammation in microglia and protective effects on hippocampal neurons. *C. annuum* leaves can be eaten raw, used to make salads, or used to make various dishes containing meat. Thus, this plant has the potential to be used as a functional food for regulating NDs.

## Figures and Tables

**Table 1:** Passport data of the 40 genetic resources from *Capsicum* spp.

Classification	Accession	Name	Species	Origin	IT Number
Breeding material	23OM01	20AGI022	*Capsicum annuum*	NIHHS	
	23OM02	20AGI024	*Capsicum annuum*	NIHHS	
	23OM03	20AGI029	*Capsicum annuum*	NIHHS	
	23OM04	Peru-5438	*Capsicum chinense*	NIAS	284050
	23OM05	C04776	*Capsicum frutescens*	NIAS	264142
	23OM06	20AGI039	*Capsicum annuum*	NIHHS	
	23OM07	Geumneung Jaerae	*Capsicum annuum*	NIAS	32454
	23OM08	Ttungttungcho	*Capsicum annuum*	NIAS	236435
	23OM09	Sarospataki tf.	*Capsicum annuum*	NIAS	237569
	23OM10	Procede-9927	*Capsicum frutescens*	NIAS	289309
	23OM11	20AGI018	*Capsicum annuum*	NIHHS	
	23OM12	20AGI001	*Capsicum annuum*	NIHHS	
	23OM13	20AGI020	*Capsicum annuum*	NIHHS	
	23OM14	20AGI021	*Capsicum annuum*	NIHHS	
	23OM15	Daejeon Jaerae	*Capsicum annuum*	NIAS	229662
	23OM16	Buyeosamseong	*Capsicum annuum*	NIAS	286355
	23OM17	Seonghwansinbang	*Capsicum annuum*	NIAS	286378
	23OM18	Uiryeonggabeul	*Capsicum annuum*	NIAS	286389
	23OM19	Gochangjusan	*Capsicum annuum*	NIAS	286392
	23OM20	20AGI049	*Capsicum annuum*	NIHHS	
	23OM21	Taean	*Capsicum annuum*	NIHHS	
	23OM22	YCM334	*Capsicum annuum*	NIHHS	
	23OM23	TF68	*Capsicum annuum*	NIHHS	
	23OM24	Habanero	*Capsicum chinense*	NIHHS	
	23OM25	35009	*Capsicum annuum*	NIHHS	
	23OM26	35001	*Capsicum annuum*	NIHHS	
	23OM27	SR213	*Capsicum annuum*	NIHHS	
	23OM28	SR214	*Capsicum annuum*	NIHHS	
	23OM29	Hanuri	*Capsicum annuum*	NIHHS	
	23OM30	Toktok	*Capsicum annuum*	NIHHS	
	23OM31	Start07	*Capsicum annuum*	NIHHS	
	23OM32	AVDan1ho	*Capsicum annuum*	NIHHS	
	23OM33	Jolokia	*Capsicum chinense*	NIHHS	
Commercial cultivar	23OM34	Kallatan	*Capsicum annuum*	Sakata Korea	
	23OM35	Gilsang	*Capsicum annuum*	Sakata Korea	
	23OM36	Nokgwang	*Capsicum annuum*	Pamhannong	
	23OM37	Cheongyang	*Capsicum annuum*	Pamhannong	
	23OM38	Dangjo Mild	*Capsicum annuum*	Jeil Seed	
	23OM39	Miin Putgochu	*Capsicum annuum*	Asia Seed	
	23OM40	Wongi1ho	*Capsicum annuum*	NIHHS	

**Table 2:** Major morphological characteristics of the 40 genetic resources from *Capsicum* spp.

	Plant Height (cm)	1st Node Length (cm)	Stem Diameter (mm)	Leaf Length (cm)	Leaf Width (cm)
23OM01	83.6 ± 18.0	34.2 ± 5.0	12.7 ± 2.3	14.5 ± 3.3	5.3 ± 1.2
23OM02	75.6 ± 25.8	35.1 ± 1.1	11.2 ± 3.8	13.8 ± 3.8	5.3 ± 1.5
23OM03	71.4 ± 4.3	31.7 ± 1.4	12.5 ± 0.6	15.8 ± 1.2	6.3 ± 0.7
23OM04	46.7 ± 5.4	14.4 ± 3.0	11.3 ± 1.7	9.5 ± 0.9	4.8 ± 0.6
23OM05	73.1 ± 6.7	36.5 ± 2.8	11.3 ± 1.4	17.6 ± 1.5	10.3 ± 1.0
23OM06	64.2 ± 18.5	18.7 ± 2.5	11.6 ± 3.3	9.1 ± 1.6	3.8 ± 0.6
23OM07	79.6 ± 26.4	25.0 ± 4.8	8.5 ± 2.3	8.3 ± 2.1	3.6 ± 0.9
23OM08	71.3 ± 10.0	16.4 ± 4.1	9.7 ± 1.5	6.7 ± 1.0	3.0 ± 0.4
23OM09	61.8 ± 14.2	27.0 ± 2.0	13.4 ± 3.2	15.1 ± 4.2	6.5 ± 1.8
23OM10	56.9 ± 4.5	33.0 ± 1.8	10.7 ± 1.1	16.3 ± 1.5	8.5 ± 1.2
23OM11	79.0 ± 11.1	27.1 ± 1.4	12.8 ± 1.1	9.9 ± 1.0	4.1 ± 0.4
23OM12	72.8 ± 8.1	28.8 ± 2.8	11.9 ± 0.8	11.2 ± 0.7	4.8 ± 0.4
23OM13	86.8 ± 4.8	33.2 ± 2.9	12.8 ± 0.8	15.4 ± 0.6	6.0 ± 0.5
23OM14	89.9 ± 7.0	35.0 ± 1.1	12.6 ± 0.7	15.9 ± 1.5	6.0 ± 0.5
23OM15	97.9 ± 9.0	30.5 ± 2.4	12.2 ± 1.0	14.7 ± 0.8	6.7 ± 0.6
23OM16	90.8 ± 12.5	29.5 ± 4.5	9.5 ± 0.5	8.6 ± 1.2	3.7 ± 0.6
23OM17	94.1 ± 7.2	26.4 ± 4.4	11.2 ± 0.9	8.8 ± 1.3	3.8 ± 0.5
23OM18	90.9 ± 6.5	32.3 ± 2.3	11.5 ± 0.7	11.1 ± 1.4	4.6 ± 0.8
23OM19	91.9 ± 10.6	26.8 ± 2.4	10.6 ± 1.0	8.5 ± 0.4	4.0 ± 0.2
23OM20	39.3 ± 5.3	16.8 ± 2.6	11.3 ± 0.8	14.5 ± 1.1	6.0 ± 0.4
23OM21	85.3 ± 18.9	25.6 ± 3.1	11.0 ± 2.4	10.1 ± 1.8	4.6 ± 0.9
23OM22	72.8 ± 7.2	27.5 ± 2.3	10.7 ± 1.0	12.9 ± 1.3	5.6 ± 0.6
23OM23	76.7 ± 20.0	26.5 ± 3.8	10.3 ± 2.0	13.7 ± 2.9	5.3 ± 1.2
23OM24	47.6 ± 3.4	22.9 ± 2.5	10.7 ± 0.8	12.3 ± 0.9	7.2 ± 0.5
23OM25	80.8 ± 6.7	23.4 ± 1.8	14.6 ± 1.3	9.0 ± 1.4	4.4 ± 1.0
23OM26	38.3 ± 11.4	22.9 ± 4.6	7.9 ± 1.2	9.6 ± 0.9	4.6 ± 0.4
23OM27	70.3 ± 4.1	24.5 ± 2.5	11.7 ± 1.0	13.6 ± 1.6	5.0 ± 0.4
23OM28	76.1 ± 7.2	31.2 ± 2.3	11.5 ± 0.5	13.6 ± 0.8	5.2 ± 0.5
23OM29	40.5 ± 2.9	14.0±1.2	6.3 ± 0.6	4.8 ± 0.3	2.1 ± 0.4
23OM30	42.6 ± 13.3	12.5 ± 2.5	10.3 ± 1.0	8.9 ± 1.1	5.5 ± 0.6
23OM31	67.5±12.3	22.5 ± 2.8	12.9 ± 1.5	13.6 ± 2.0	6.2 ± 0.8
23OM32	51.0 ± 11.6	24.8 ± 1.8	13.3 ± 2.9	14.6 ± 4.1	6.5 ± 1.5
23OM33	64.3 ± 8.9	28.0 ± 6.8	11.9 ± 0.8	15.3 ± 2.3	9.0 ± 1.4
23OM34	91.5 ± 8.0	26.4 ± 2.4	12.8 ± 1.8	10.2 ± 1.9	4.5 ± 0.8
23OM35	71.1 ± 4.4	21.9 ± 2.1	12.4 ± 0.9	10.2 ± 1.2	4.5 ± 0.6
23OM36	91.3 ± 9.6	27.2 ± 2.1	12.8 ± 1.3	12.0 ± 1.8	5.3 ± 0.9
23OM37	111.6 ± 4.0	29.7 ± 2.4	14.7 ± 1.1	9.7 ± 1.1	4.2 ± 0.5
23OM38	71.0 ± 6.7	30.8 ± 0.7	13.7 ± 0.9	14.9 ± 2.2	6.6 ± 1.1
23OM39	76.0 ± 4.0	22.6 ± 1.8	13.2 ± 0.8	10.4 ± 1.0	4.1 ± 0.7
23OM40	77.3 ± 6.2	28.6 ± 4.0	12.3 ± 1.2	11.8 ± 0.8	5.7 ± 0.4

**Table 3:** Cell protection EC50 of 23OM16-23OM20 on glutamate-induced HT22 cells.

Accession	Name	Cell Protection EC_50_ (μg/mL)
23OM16	Buyeosamseong	167.5 ± 0.8095
23OM17	Seonghwansinbang	166.4 ± 0.8032
23OM18	Uiryeonggabeul	102.6 ± 0.2934
23OM19	Gochangjusan	147.4 ± 1.109
23OM20	20AGI049	167.2 ± 8.047

**Table 4:** Relative percentages of secondary metabolite in four genetic resources from *Capsicum* spp. (%).

No.	Expected RT (min)	Component Name	Formula	Observed *m*/*z*	Observed RT (min)	Adducts	Average of Relative Percentage (%)				
							OM-16	OM-17	OM-18	OM-19	OM-20
1	0.59	Quinic acid	C_7_H_12_O_6_	191.01	0.63	−H	8.017	6.410	6.884	9.507	10.328
2	0.79	Citric acid	C_6_H_8_O_7_	191.05	0.8	−H	4.507	5.132	3.811	4.797	3.265
3	0.92	N-caffeoylputrescine	C_13_H_18_N_2_O_3_	249.12	0.91	−H, +HCOO	7.321	2.391	4.287	4.299	4.965
4	1.53	Chlorogenic acid	C_16_H_18_O_9_	353.09	1.5	-H	2.286	4.052	1.822	4.136	1.573
5	2.48	Maltose	C_12_H_22_O_11_	341.09	2.39	−H, +HCOO	0.006	N.D	N.D	N.D	N.D
6	2.81	Luteolin-7-*O*-apiofuranosyl(1→2) glucopyranoside	C_26_H_28_O_15_	579.13	2.82	−H, +HCOO	27.149	25.186	26.800	26.064	24.708
7	2.91	Luteolin-7-*O*-glucopyranoside	C_21_H_20_O_11_	447.09	2.91	−H, +HCOO	12.057	6.826	11.895	11.319	10.833
8	3.31	Apigenin-7-apiosylglucoside	C_26_H_28_O_14_	563.14	3.3	−H, +HCOO	2.188	19.412	2.190	1.167	5.491
9	3.39	Luteolin7-(2-*O*-apiosyl-6-*O*-malonyl) glucoside	C_29_H_30_O_18_	665.14	3.39	−H	18.308	12.835	16.887	21.861	17.799
10	3.52	Luteolin 7-0-β-d-(6″-acetyl)-glucopyranoside	C_23_H_22_O_12_	489.1	3.51	−H	3.516	1.184	2.769	4.658	4.085
11	3.9	Malonylapiin	C_29_H_30_O_17_	649.14	3.89	−H	0.466	3.346	0.374	0.486	1.73
12	5.13	Capsianoside III	C_50_H_84_O_26_	1145.52	5.14	−H, +HCOO	0.547	0.809	0.735	0.367	0.950
13	5.67	Capsianoside I	C_32_H_52_O_14_	659.33	5.68	−H	0.484	0.720	0.666	0.592	1.616
14	5.94	Capsianoside II	C_50_H_84_O_25_	1129.53	5.95	−H, +HCOO	7.414	7.447	11.632	4.559	7.090
15	6.37	Capsianoside new III	C_53_H_86_O_28_	1169.53	6.36	−H	5.567	3.955	9.057	6.109	5.107
16	10.86	Capsianoside VIII	C_50_H_84_O_25_	1129.53	10.85	−H, +HCOO	0.169	0.297	0.192	0.079	0.487

## Data Availability

The data are contained within the article.
